# Analysis of death in major trauma: value of prompt post mortem computed tomography (pmCT) in comparison to office hour autopsy

**DOI:** 10.1186/s13049-016-0231-6

**Published:** 2016-03-29

**Authors:** MArkus Schmitt-Sody, Stefanie Kurz, MAximilian REiser, Karl Georg Kanz, Chlodwig Kirchhoff, Oliver Peschel, Sonja Kirchhoff

**Affiliations:** Klinikum der Universitat Munchen, Munich, Germany

## Abstract

**Background:**

To analyze diagnostic accuracy of prompt post mortem Computed Tomography (pmCT) in determining causes of death in patients who died during trauma room management and to compare the results to gold standard autopsy during office hours.

**Methods:**

Multiple injured patients who died during trauma room care were enrolled. PmCT was performed immediately followed by autopsy during office hours. PmCT and autopsy were analyzed primarily regarding pmCT ability to find causes of death and secondarily to define exact causes of death including accurate anatomic localizations. For the secondary analysis data was divided in group-I with equal results of pmCT and autopsy, group-II with autopsy providing superior results and group-III with pmCT providing superior information contributing to but not majorly causing death.

**Results:**

Seventeen multiple trauma patients were enrolled. Since multiple trauma patients were enrolled more injuries than patients are provided. Eight patients sustained deadly head injuries (47.1 %), 11 chest (64.7 %), 4 skeletal system (23.5 %) injuries and one patient drowned (5.8 %). Primary analysis revealed in 16/17 patients (94.1 %) causes of death in accordance with autopsy. Secondary analysis revealed in 9/17 cases (group-I) good agreement of autopsy and pmCT. In seven cases autopsy provided superior results (group-II) whereas in 1 case pmCT found more information (group-III).

**Discussion:**

The presented work studied the diagnostic value of pmCT in defining causes of death in comparison to standard autopsy. Primary analysis revealed that in 94.1% of cases pmCT was able to define causes of death even if only indirect signs were present. Secondary analysis showed that pmCT and autopsy showed equal results regarding causes of death in 52.9%.

**Conclusions:**

PmCT is useful in traumatic death allowing for an immediate identification of causes of death and providing detailed information on bony lesions, brain injuries and gas formations. It is advisable to conduct pmCT especially in cases without consent to autopsy to gain information about possible causes of death and to rule out possible clinical errors.

## Background

Trauma is the leading cause of death in young adults between 15 and 35 years [1, 2]. According to the German trauma registry 5.2 % of all multiple injured patients decease already during the early posttraumatic phase while undergoing trauma room management [3]. Therefore further analysis is necessary to clarify causes of death, particularly in case of unsuccessful resuscitation efforts. In this context a critical review of literature revealed that autopsy proved approximately 30 % of clinically defined causes of death as wrong [4–7]. Although autopsy is still the gold standard of postmortem examination, its number has significantly decreased over the years due to emotional, legal or religious reasons [4]. On this account non-invasive postmortem exams gain in clinical importance. In this regard Postmortem computed tomography (pmCT) has been shown by several authors to be a promising technique for either supplementing autopsy or, in case of declined autopsy, replacing it. However, a stringent analysis of the diagnostic value of pmCT in comparison to standard autopsy in defining potential causes of death in patients who died during the early posttraumatic phase has yet not been performed.

Therefore the aim of this study was to analyze pmCT fndings and its ability in primarily finding causes of death and secondarily defining death-related diagnoses in multiple trauma patients deceased during trauma room management in comparison to autopsy especially in cases of unsuccessful resuscitation efforts to possibly rule out additional clinical maltreatment.

## Methods

The study was approved by the local board of ethics (reference-nr 151-08).

### Subjects

The primary inclusion criteria was admittance to our Level-I-trauma-center between 1999 and 2007 due to major trauma with a consecutive need of resuscitation and declaration of death in consensus by the trauma team. Another inclusion criteria was the performance of autopsy following pmCT during office hours. Also the corresponding autopsy reports as well as the medical charts needed to be available for the analysis.

### Postmortem computed tomography (pmCT)

Corpses of the deceased trauma-patients underwent CT 5 to 60 min after death was declared. CT without intravenous contrast agent was performed with all inserted medical equipment e.g. endotracheal tubes, central lines, left in place. From 1999 until 2005 CT was acquired on a 4-slice-CT-scanner (Proven-Excellence, Somatom-Sensation-4, Siemens-Medical-Solutions, Germany) and from 2006 to 2007 on a 64-multi-slice-scanner (LightSpeed VCT 64, GE-Healthcare Massachusetts, USA).

The pmCT-exam comprised a CT of the head, the entire spine, thorax, abdomen including pelvis and proximal upper thighs so that the pmCT was comparable to a multiple trauma CT scan, allowing thereby an optimal comparability since in many cases a multiple trauma CT was performed earlier in the diagnostic trauma room workup.

For performing head CT a slice thickness of 2.5 mm was used on the 64-slice scanner, 2 and 5 mm respectively on the 4-slice scanner using a sequential protocol. Regarding CT exams of thorax and abdomen on the 64-slice scanner a slice thickness of 1.25 mm was used compared to a slice thickness of 5 mm on the 4-slice scanner.

PmCT-data was evaluated in a consensus-reading by one expert forensic board-certified radiology fellow and one board-certified forensic pathology fellow whereas both modalities were blinded against each other. The evaluating radiologist was blinded to clinical as well as to autopsy data.

### Autopsy

Autopsy was performed according to common standards of the German Government guidelines (§87,89 German-Code-of-Criminal-Procedure). Subjects were examined at our University Institute of Legal Medicine 2 to 98 h after death during office hours. Autopsy-data were retrospectively analysed.

### Comparison pmCT-autopsy

Cause of death was defined as one or more combined injuries not accordable with life assigned to one organ or organ-system e.g. brain-stem incarceration due to intracranial hemorrhage and consecutively increased intracranial pressure.

#### Primary analysis

Causes of death defined in autopsy were recorded and compared in detail to pmCT-results for agreement. For the primary analysis potential causes of death determined by pmCT were considered as true-positive even if the definition of the exact anatomic region attributable to the cause of death was not definitely possible e.g. huge amount of blood within the chest defined as bleed-to-death due to vascular lesions not exactly pinpointable on pmCT.

#### Secondary analysis

For the secondary analysis data was divided into group-I (matching diagnoses) revealing equal findings in pmCT and autopsy regarding cause of death as well as its anatomic localization, in group-II (overlapping diagnoses) where autopsy provided more information regarding the exact cause of death and its anatomic localization and in group-III (death-related diagnoses) where pmCT provided additional information regarding diagnosis contributing to but not majorly causing death.

In addition, pmCT and autopsy were evaluated for other findings besides causes of death. These findings were recorded and compared to be able to carve out additional advantages of pmCT.

### Data-analysis

The inter-reader agreement of causes of death in autopsy and pmCT respectively was determined using the Cohen-*K*-test as statistical measure of inter-reader agreement for qualitative items.

## Results

### Subjects

In the study period between the years 1999 and 2007 overall 55 patients died for any reason in the trauma room of our level I University Trauma center. According to our inclusion criteria only patients who were admitted for major trauma with the need of resuscitation either in the trauma room or during emergency surgery being terminated on a consensus decision of the trauma team were enrolled. Since the presented work is a comparison study between PMCT and autopsy another inclusion criteria was the performance of autopsy and consequently available forensic as well as clinical chart and data respectively. Therefore only the small number of 17 patients met our inclusion criteria over this rather long study period.

Seventeen patients were enrolled (12 (71 %): male; 5 (29 %): female) with a mean age of 41.7 years (age-range 23–68 years). Eleven (65 %) patients sustained traffic accidents, two (12 %) had downfalls, one (6 %) sustained a train-accident and three (18 %) patients suffered from other accidents. Regarding emergency-procedures performed after admittance to the trauma-room 14 patients underwent CPR whereas in eight cases CPR was performed along with thoracotomy and even open heart massage (*n* = 6). Emergency-laparotomy was performed in six patients and another two patients received external ventricular drainage. All patients deceased in the trauma-room whereas six patients were admitted under resuscitation.

PMCT examinations in the years 1999 until 2005 were performed on a 4-slice CT scanner so that the experience of analyzing pmCT images was higher compared to an only two years examination-experience of the 64-slice scanner. However, the accuracy of the diagnoses was quite higher on the 64-slice scanner especially regarding pathologies of the parenchymal organs due to improved scanning parameters. Though, there was no statistical significant difference regarding sensitivity and specificity regarding pmCT diagnoses detectable.

### Cause-of-death - autopsy

Since multiple trauma patients were enrolled more injuries than patients are described. Eight patients sustained deadly head injuries (47.1 %), 11 thoracic (64.7 %), 4 skeletal system-injuries (23.5 %) and 1 patient drowned (5.8 %).

### Primary analysis

PmCT found in 16/17 patients (94.1 %) causes-of-death comparable to autopsy. Only one patient presented no pathological pmCT-findings whereas autopsy defined chest as region- and suffocation as cause of death.

### Secondary analysis

Overall in 52.9 % (nine cases) good agreement in autopsy and pmCT resulted (group-I, Table 1). In 41.2 % (seven cases) autopsy provided superior results compared to pmCT (group-II, Table 2) and in 5.8 % (one case) pmCT found more information than autopsy regarding diagnosis contributing to but not majorly causing death (group-III, Table 3).

**Table 1:** Overview of group I patients presenting good agreement of results in pmCT and autopsy (DD = differential diagnosis)

ID	Cause of death - pmCT	Cause of death - autopsy	Region of death causing injury
1	subtotal amputation pelvic half/thigh amputation os ilium, open book fracture	amputation pelvis with consecutive blood loss	Skeletal system
7	brain steem bleeding, incarceration foramen magnum avulsion cervical myelon (C1/2)	basal skull fracture, brain bleeding, incarceration of medulla oblongata	head/neck
8	Multiple trauma: complex dislocated fracture skull/foramen magnum, subarachnoidal bleeding, ventricle tamponade brain edema, incarceration suspected aortic root rupture, hematothorax, lung contusion, Tension pneumothorax, disruption pericardium/heart	multiple trauma: destruction of basal skull, disruption of pons/medulla oblongata, ventricle tamponade, diffuse intracerebral bleeding disruption of lung/pericardium/heart, rupture of aortic root, hematothorax	head/neck chest
13	intracerebral, subdural, subarachnoidal bleeding brain edema, basal incarceration	bloody destruction, herniation frontal/temportal lobe with so-called intravital brain necrosis	head/neck
14	impression fracture skull brain edema, incarceration lung contusion DD signs of aspiration	brain injury with occipital impression fracture, brain contusion, brain edema blood aspiration lung	head/neck chest
16	brain edema atlas ring fracture, dislocated dens axis, intraspinal bleeding avulsion vertebral artery, suspected marrow injury cervical spine	gaping disconnection 1st and 2nd cervical vertebrae, atlas ring fracture with rupture upper cervical marrow	head/neck
18	brain edema due to oxygen lack (drowning) lung edema, fluid filled major airways	signs of drowning lung/distal airways brain edema	chest
22	bilateral rib fractures pleural effusion, bilateral hematothorax	bilateral rib fractures, hematothorax, pleural effusion with circulatory failure due to inner bleed to death	chest
26	signs of increased intracranial pressure, midline shiftintracranial bleeding, incarceration foramen magnum skull base fracture bony destruction pelvis	skull base fracture, subdural bleeding, signs of increased intracranial pressure, beginning incarceration complex destruction of bony pelvis, soft tissue hematoma retroperitoneal due to rupture internal iliac artery	head abdomen/pelvis

**Table 2:** Overview of group II patients presenting superior results in autopsy (detailed cause of death found in autopsy is marked bold)

ID	Cause of death - autopsy	Cause of death - pmCT	Region of death causing injury
**2**	**Distal thoracic aortic rupture,** Rupture of bronchus/azygos vein	pleural effusion/hematothorax = signs of bleed to death	chest
**3**	Multiple trauma/inner bleed to death **rupture iliac artery/inner organs, open pericardium** lung contusions, pneumthorax with pleural effusion fractured pelvis, retroperitoneal hematoma brain edema with incarceration	suspected rupture iliac artery free intraabdominal fluid = signs of bleed to death lung contusion/pneumothorax, pleural effusion brain edema with upper and lower incarceration	skeletal system Head/neck Chest
**6**	**Chest compression** most likely suffocation	no directly death causing injury reported	chest
**19**	Multiple trauma/circulatory collapse **total rupture descending aortic arch, rupture pericardium **bilateral pneumothorax, bloody pleural,effusion, pneumomediastinum serial rib fractures, bony injury pelvis, spine laceration of liver, rupture spleen	suspected rupture aorta, not definitely recognized, pneumopericardium bilateral pneumothorax, bloody pleural effusion, pneumomediastinum serial rib fractures, bony injury pelvis, spine laceration of liver, rupture spleen	chest
**20**	Multiple trauma/inner bleed to death: complex fractures spine, pelvis, sacrum **rupture pulmonary root,** lung contusion, pneumothorax bilateral serial rib fractures **rupture renal capsula/urinary bladder, retroperitoneal hematoma extensive soft tissue damage/bleeding**	complex fractures spine, pelvis, sacrum lung contusion, pneumothorax bilateral serial rib fractures retroperitoneal hematoma free intraabdominal fluid = signs of bleed to death	Skeletal system abdomen
25	Multiple trauma: brain edema, disruption articulation skull base-atlas **damage to cervical spinal cord/medulla** sub- total rupture ascending aorta with bleeding soft tissue neck mediastinum, pericardium with bilateral hematothorax pelvis fracture with soft tissue bleeding	brain edema, disruption articulation skull base-atlas total rupture ascending aorta with bleeding soft tissue neck/mediastinum, pericardium with bilateral hematothorax pelvis fracture with soft tissue bleeding	Head/neck chest
29	**Thoracic aortic rupture **status post open cardiac massage	hematothorax = signs of bleed to death status post open cardiac massage with serial rib fractures	chest

**Table 3:** Overview of group III patients with superior results on pmCT (additional findings on pmCT contributing to death but not majorly causing is marked bold)

ID	Cause of death - pmCT	Cause of death - autopsy	Region causing death
24	fracture orbita, midface **fracture dens axis with displaced fragment into spinal canal** disruption pericardium with pericardial tamponade lung contusion, bilateral pneumothorax, blood occluded airways, serial rib fractures laceration liver displaced hip joint/fracture acetabulum	fracture orbita, midface disruption pericardium with pericardial tamponade lung contusion, bilateral pneumothorax, blood serial rib fractures, lung disruption, soft tissue bleeding laceration liver	Skeletal system chest

#### Group-I - matching diagnoses

**Fig. 1:**
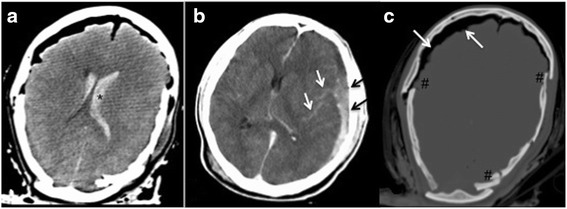
The soft-tissue-window of a cerebral CT of a young patient demonstrates the advantages of pmCT in terms of clearly showing intraventricular hemorrhage (**a**,*), subarachnoidal bleeding (see *white arrows*) along with subdural hematoma (see *black arrows*) on the left side (see (**b**) without changing the pressure-in situ. The display of CT in the bone window additionally demonstrates the multiple fragment fracture (#) of the calvarium as well as massive free intracranial air (see *white arrows*) (**c**)

66.7% of group-I-patients showed injuries to the head/neck-region, 44.4 % were thoracic pathologies. Deadly injuries of the skeletal-system were diagnosed in 33.3 % of the cases (Table 1). One patient showed fluid-filled bronchia/trachea associated with signs of severe lung- but also brain-edema on CT. Knowing the fatal incident the diagnosis drowning was stated based on pmCT-findings and confirmed by autopsy. In case ID8 pmCT considered pleural effusion and hematothorax as indirect signs of bleed-to-death, thus cause of death was considered true-positive in the primary analysis. It was not possible to state the direct diagnosis of an aortic rupture and exact anatomic localization respectively on pmCT. However, this patient was included in group-I since he died not only due to thoracic pathologies but due to a combination of head/neck and chest-pathologies correctly diagnosed on pmCT (Table 1) (see Fig. 1).

#### Group-II overlapping diagnoses

In all cases enrolled in group II injuries to the thoracic region were considered as cause of death. In the majority of cases (57.1 %) rupture of the aorta was determined as cause of death.

In two cases additional skeletal injuries and in another two cases additional head/neck injuries were relevant for the cause of death (Table 2).

**Fig. 2:**
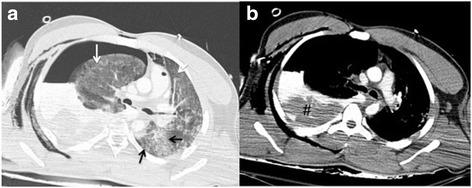
CT-images of the chest after falling from a great height demonstrate in the lung window (**a**) extensive lung parenchymal lacerations (see *black arrows*) along with contusions of the lung (see *white arrows*). Pleural effusion (soft tissue window, **b** demonstrates blood-like density values along with condensed portions in terms of hematothorax (see #) and corresponding mediastinal shift towards the left side. However, the exact source of bleeding was only suspected and evaluated as bleed to death but not exactly detected on pmCT but stated by the gold standard autopsy in terms of aortic rupture as cause of death

PmCT of one case showed a hematothorax suggesting a rupture of a lung bronchus or of the azygos vein but it was not possible to identify the exact source of bleeding on pmCT. Even on antemortem-CT with contrast agent no source of bleeding was described and detected respectively. In this specific case autopsy revealed a rupture of the thoracic aorta as cause of death (ID2) (see Fig. 2). In another two cases, hematothorax was described on pmCT and considered as indirect sign of a rupture of a large vessel, but the final diagnosis was only possible in autopsy (ID19, 29). In one case antemortem CT images demonstrated a rupture of the aorta (ID25), however these findings were not detected on pmCT.

During resuscitation one case (ID6) underwent chest compression antemortem. However, in this case pmCT did not reveal any cause of death, but autopsy described suffocation as cause of death.

#### Group-III death-related diagnoses

**Fig. 3:**
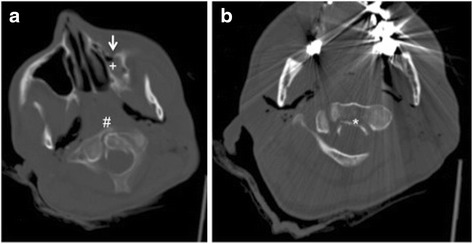
CT of the cervial spine shows a type II dens fracture (#) with a dorsal dislocation of a fragment (*) towards the spinal canal (**b**). **a** also demonstrates an additional fracture of the left maxillary sinus (see *white arrow*) filled with blood (+) as another example of the great potential of diagnosing injuries to the bone especially of the face using CT

PmCT reveald in this one case enrolled in group III a dislocated fracture of the second cervical vertebra. However this diagnosis was not described in autopsy. Overall this pmCT finding only contributed to death, but was not the relevant finding in terms of cause of death (see Fig. 3).

## Discussion

The diagnostic value of pmCT in defining the cause of death in comparison to standard autopsy was studied. The primary analysis in terms of finding causes of death even if only indirect signs e.g. of bleed-to-death, were present, revealed that it is possible to define reliable causes of death on pmCT in 94.1 %. The secondary analysis comprised pmCT’s ability to define exact causes of death including the exact anatomic localization in 52.9 %. In parallel autopsy yielded a distinct cause of death mostly due to parenchymal organ or vessel pathologies in 41.2 %. In contrast pmCT found more death-related information in 5.8 %. Therefore pmCT seem to be a useful diagnostic adjunct to unsuccsessful trauma room management enabling for immediate identification of cause of death and detailed information on skeletal and cerebral injuries, but also on gas formations.

In addition all dis- and advantages of both modalities were recorded. PmCT was superior to autopsy in diagnosing skeletal injuries and displaying pressure-in-situ (e.g. pneumothorax). As known from the literature, but also recognizable from our results, pmCT displayed cerebral pathologies well. However, native pmCT is less valuable in diagnosing parenchymal organs’ or vascular pathologies considered as autopsy’s major advantage.

In this context Hoey et al. evaluated [8] pmCT for finding causes of death in trauma patients. The authors analysed 12 patients with consecutive limited validity and transferability to a broader patient collective. Causes of death were divided in blunt head-, chest- and abdominal-trauma. A more detailed injury-analysis as presented allows for a deeper understanding of pmCT-abilities finding reliable causes of death. The exact differentiation of topography and injury-type is crucial since CT provides different diagnostic ability depending on the body region and tissue to be examined.

Christe et al. [9] focused on sensitivity and specificity of pmCT in 34 abdominal trauma cases in comparison to autopsy. Comparable to our results the authors concluded that small organ injuries seem undetectable by CT leading to rather low sensitivity, but nevertheless most of the life-threatening liver-injuries considered as essential in forensic pathology were detected.

### Results

#### Group-I matching diagnoses

The predominant causes of death were injuries to the head and skeletal injuries mostly of the bony pelvis along with soft tissue damage and severe bleeding.

- Skeletal injuries
  Detection of bony pathologies especially fractures is excellent on pmCT comparable to the assessment of bones in clinical radiology [10, 8, 11, 12]. In addition, pmCT is able to diagnose other skeletal injuries as stated in the literature [5, 7, 13–16]. The comparison of injury-patterns in the head/neck-region as cause of death was in 41.2 % equivalent in pmCT and autopsy. This is particularly relevant considering the increased mortality of head injuries in trauma patients [15]. However, some studies showed disadvantages of CT in detecting fractures with the exception of dislocated fractures or surrounding bleeding [10, 11]. However, in most of the presented cases fractures were either dislocated or surrounded by blood, so that these observations can be neglected.
  In addition, fracture-diagnosis in regions difficult to reach in autopsy, e.g. pelvis or cervical spine, was superior on pmCT [17]. Examining these regions in autopsy is substantially more complicated and also only possible in a destructive manner than interpreting CT-scans [18], therefore autopsy of e.g. the facial bones is only performed if certain evidence is given.
- Brain injuries
  The presented results show the great ability of pmCT to diagnose traumatic pathologies of the brain. However, Jacobsen et al. found out that small bleedings and contusions were often not displayed on pmCT [10]. However, our study-focus was the definition of causes of death whereas such small brain lesions are irrelevant and do not account for causes of death. Autopsy and pmCT were equal in diagnosing subdural, subarachnoidal hemorrhage and cerebral laceration [10] whereas CT was superior in identifying epi- and subdural hemorrhage [17], but also in detecting contusions. For diagnosing increased intracranial pressure CT is very-well eligible, since the brain is examined noninvasively without changing the pressure-ratio [19, 11].
- Postmortem changes
  In general for the evaluation of pmCT signs of decomposition always need to be considered. Possible microscopical postmortem changes are already present at the time of imaging but not yet explicitly detectable by CT, therefore not relevant for misdiagnosis or false-negative findings regarding the cause of death. Certain influence of the type of death is known on the formation of some postmortem phenomena, such as the development of cerebral gas embolization after resuscitation [20]. However, in general further research to define these and other artifacts is still necessary.
- Group-II Overlapping diagnoses
  Especially injuries of great vessels are difficult to diagnose on native CT. This limitation of pmCT is crucial, since vascular injuries form the second most common cause of death [21, 16, 22]. However, this limitation might be overcome by performing postmortem angiography, evaluated by Ross and Grabherr et al. substituting the missing circulation by a heart-lung-machine [23]. In most group II cases diagnosis of hematothorax was considered as indirect sign of bleeding as cause of death in the primary analysis. However, the definite diagnosis including the exact localization was only provided in autopsy or if contrast-enhanced antemortem CT was available for comparison.
  Rib fractures are well displayed on CT, particularly if dislocated or with surrounding bleeding [11]. Furthermore, pmCT provides an overview of the injury-system and type as well as direction of impact [24]. Pneumothorax and tension-pneumothorax are very well displayed on pmCT. CT is also superior to autopsy in finding gas formations also applying to air embolism, air in the spinal canal and soft tissue emphysema [17, 21, 16, 25].
  Postmortem-changes of the lung crucially depend on the time of examination after death. According to Stein et al. [26] these changes complicate finding causes of death. Already two hours after death, the formation of inner livor mortis begins. CT will show a densification of the lung parenchyma [27]. Particularly in trauma-patients, antemortem multiple trauma CT-scans can help to differentiate postmortem changes from injuries, such as lung hemorrhage or contusion. All in all postmortem-changes and their presentation in imaging need further research as mentioned above.
- Group-III death-related diagnoses
  PmCT of the only group III case demonstrates a dens-fracture type III (AndersonD’Alonzo), with discrete dorsal dislocation and fragment protrusion into the spinal canal. This case presents the distinct advantages of pmCT in easily evaluating body regions such as the cervical spine compared to autopsy. Available three-dimensional display allow for an easy assessment of dislocation [28]. It is shown in general that additional CT-scans before autopsy can help to rule out eventualities, since the injury pattern and its impact is not always well predictable. Scholing et al. described that CT displayed a number of injuries not found in autopsy [2].

### Limitations

PmCT is a non-invasive valuable imaging-technique with only decades of experience. Autopsy owns hundreds years of experience and an unlimited field of examination. However, several body regions are very difficult to access and in a destructive manner, so that pathologies in these regions are possibly missed if no indirect signs or certain evidence is [17]. Most presented multiple trauma- and pmCT data were acquired on a 4-slice-CT-scanner with a broader layer thickness compared to CT-scanner of newer generations. Thus, pmCT-findings might be worse compared to autopsy especially regarding small brain pathologies. In this context the resolution of the CT-scanner plays an important role as stated in the literature [17].

Since the radiologists evaluating pmCT were aware of the findings of antemortem multiple trauma CT scans a certain bias in finding additional information on pmCT can not be excluded.

In this context a minor limitation is the relatively small number of patients enrolled, the descriptive analysis performed and its retrospective character.

In general trauma room care is managed by the trauma-team consisting of one surgery-, one anesthesiology- and one radiology fellow. The trauma team reaches consensus decisions regarding diagnostics and therapy, but also regarding the performance of pmCT as actually presented. The primary endpoint for performing pmCT was the possibility to confirm/detect causes of death, whereas the secondary endpoint was the possiblity to find missed but treatable injuries. Otherwise if a multiple trauma patient died due to unknown or not obvious reasons the trauma team would go home with potentially evolving emotional stress since they would not have been certain they had done everything possible to save the patient’s life.

## Conclusion

Due to decreasing numbers of autopsies and technical development in radiology and CT especially, the possibility of finding causes of death by pmCT was studied. PmCT is useful in trauma victims who died in the trauma room providing immediate identification of causes of death and detailed information on especially bony lesions, but also of brain injuries and gas formations. In 16/17 patients pmCT was able to find reliable causes of death right after death was declared. However, a more detailed analysis showed that in 10/17 cases it was also possible to find the exact and more detailed cause of death in pmCT correspondingly to autopsy, whereas autopsy revealed additional information regarding causes of death in the remaining seven cases. It is advisable to perform pmCT especially when consent to autopsy is missing to gain information about possible causes of death and to rule out the possibility of clinical errors since CT presents a noninvasive alternative to evaluate diagnosis and therapy. Thus pmCT will play an increasingly important role in legal medicine in the future.
